# Barriers to the diagnosis of somatoform disorders in primary care: protocol for a systematic review of the current status

**DOI:** 10.1186/2046-4053-2-99

**Published:** 2013-11-08

**Authors:** Alexandra M Murray, Anne Toussaint, Astrid Althaus, Bernd Löwe

**Affiliations:** 1 Department of Psychosomatic Medicine and Psychotherapy, University Medical Center Hamburg-Eppendorf, Hamburg, Germany; 2 University Hospital of Psychosomatic Medicine and Psychotherapy, Schön Clinic Hamburg-Eilbek, Hamburg, Germany

**Keywords:** Somatoform, Somatization, Somatisation, Primary care, Diagnosis, Recognition, Problems with diagnosis, Medically unexplained symptoms, Systematic review, Functional symptom

## Abstract

**Background:**

Somatoform-type disorders and functional medically unexplained symptoms are extremely common in primary care settings. These disorders, however, are consistently underdiagnosed and under-recognised which precludes effective treatment. Given that somatoform symptoms are associated with high impairment, healthcare costs and both physician and patient frustration, it is critical to improve early detection. The first step in improving patient care is to identify the current barriers which obstruct successful diagnosis to enable the design of targeted interventions. We aim to conduct a systematic review to identify the possible physician-, patient- and society-related factors and other practical constraints which may impede successful diagnosis. In the process, we will also be able to recognise the differences in methodological techniques, recommend potential avenues for future research and comment on the literature in this field as a whole.

**Methods/Design:**

We aim to conduct a systematic review of the relevant peer-reviewed literature published in English or German in the past 10 years in MEDLINE, EMBASE, PsycINFO and the Cochrane Database of Systematic Reviews. Additional studies may be identified from the reference lists of included studies. Title and abstract screening and data extraction from full text manuscripts will be conducted by two independent reviewers. Because we are including a combination of qualitative and quantitative studies, the review will provide a broad understanding of the current situation. Wherever possible, the method and reporting of the review will adhere to the guidelines outlined in the PRISMA statement and bias will be assessed using the Cochrane collaboration’s recommendations. We envisage that data will be synthesised using a multilevel (qualitative and quantitative) approach which combines textual narrative and thematic analysis. Barriers will be categorised as modifiable or non-modifiable according to a conceptual framework. The review has been registered in an international registry of systematic reviews PROSPERO (CRD42013002540).

**Discussion:**

We hope that this study will provide an insight into the barriers to diagnosis of somatoform-type disorders and the results can be used to target appropriate interventions to improve care for these patients.

## Background

### Somatoform disorders in primary care

Symptoms which do not have a 'medical’ explanation are extremely common in primary care [1,2] and are central to the diagnosis of somatoform-type disorders when they are associated with psychological distress and high healthcare use [3]. Prevalence rates for these disorders have shown to be between 16.1% and 57.5%^a^ of primary care patients [4-6] with a 12-month prevalence of 11% in the general population [7]. Not only are these disorders common, they are expensive: the estimated cost of managing patients with somatoform disorders in Europe in 2010 was €21.2 billion [8]. However, the successful detection of somatoform disorders in primary care is not only important to reduce healthcare costs but also to improve patient care; somatoform disorders are also associated with significant impairment, especially in patients with psychiatric co-morbidity [5]. Effective methods need to be developed to ensure that patients with somatoform-type disorders are identified and treated accordingly [9,10].

Family doctors are critical to the successful detection and management of patients with somatoform-type disorders [11,12]. Primarily, this is because patients first present their symptoms to their general practitioner [1]. Being able to diagnose somatoform-type disorders is important for both doctors and patients to help conceptualise and communicate information about unexplained symptoms [13]. The correct diagnostic label can not only legitimise concerns but also enable a targeted treatment [14]. Current progress in this area is apparent, for example, the availability of evidence-based diagnostic guidelines [10].

Despite the high prevalence rates, importance and costs of such unexplained symptoms in primary care, the diagnosis of somatoform-type disorders is often either very delayed, indirect or not made at all [12]. The very nature of unexplained symptoms are often vague or difficult to characterise which makes differential diagnosis difficult [15]. This problem is exacerbated when considering the high co-morbidity of somatoform disorders with other mental disorders like depression or anxiety disorders [4,5,7]. The clear gap between the presence and diagnosis suggests that there are some barriers in the process which prevent successful diagnosis. With the correct training and tools, the successful diagnosis of somatoform disorders could be dramatically increased [16].

### Potential barriers to successful diagnosis

We propose that there are a number of potential types of barriers to the diagnosis of somatoform disorders in primary care. These barriers can be categorised into those which are modifiable and those which are not - at least without widespread systemic change (see methods for the conceptual framework). Whether or not we identify and confirm these barriers in the process of the review, however, will become apparent once the results are compiled.

Previous research suggests that there are modifiable, doctor-related factors which may impede the successful diagnosis of somatoform disorders. At least before the introduction of DSM-5 [17], the diagnosis of somatoform disorders relied on the idea that symptoms are 'medically unexplained’. It was, therefore, ultimately based on a diagnosis of exclusion [12,18,19]. Such uncertainty as to the cause of the symptoms can create unease in physicians who must balance the necessity of ruling out serious illness and increasing chronicity against the cost and distress of extensive testing [20]. Given the potential legal and professional complications, physicians are justifiably concerned about ruling out organic disease and may often focus on the physical side of the symptoms [21]. It may be this fear of missing a 'serious diagnosis’ [22] that underlies physicians’ reluctance to dismiss physical symptoms and focus on psychological distress during consultations [23,24]. Additionally, physicians may be uncomfortable with doing 'nothing’ so may intervene, even when unnecessary [23]. The problem of intervening or testing excessively, however, is that when they 'fail’ to find a cause it may result in further frustration [25]. Whether this reflects a need for further training of physicians or a paradigmatic shift in the way consultations are conducted, is still unclear.

In contrast, there may be modifiable barriers which can be attributed to patient-related factors. For example, patients may create barriers to successful diagnosis with the information they choose to disclose (or withhold); for example, there is evidence to suggest that patients are reluctant to disclose psychosocial problems in primary care settings [16,18]. This may be because they are socially embarrassed or feel as though family practice is not the appropriate setting. Regardless of the reason, this then leaves the physician to either make assumptions about the patient’s situation [16] or to develop a better patient-physician communication strategy.

From a different perspective, somatoform-type disorders are associated with some conceptual difficulties which are not so easily modifiable. The idea that western medicine is based on an inherent mind-body dualism which separates the physical from the mental [12,26] may fundamentally hinder the diagnosis of somatoform-type disorders in clinical practice. Such thinking is also related to the idea that every symptom needs an identifiable cause [25]; patients, employers, spouses and friends, therefore, all expect that there should be some underlying explanation of the patient’s symptoms. Van Staden [21], among others, suggests that such reductionist thinking can become pathological when dealing with patients with unexplained symptoms because there may be a resistance to accept purely psychological or psychiatric explanations and patients may then search for more acceptable explanations [26]. This may help explain why a surprising number of patients still continue to seek physical treatment when a diagnosis of medically unexplained symptoms is given or 'shop’ for another physician who gives a better account of their symptoms [9,18]. Not only does this become a waste of resources, but it precludes possible successful treatment and care for these patients.

Similarly to the difficult-to-modify conceptual issues surrounding somatoform disorders, there may be some barriers in the operationalisation of the diagnosis itself. During the preparation period of DSM-5, the diagnostic criteria for somatoform-type disorders have been hotly debated and discussed in the literature [19,26-29]^b^. On the one hand, some researchers suggest that the DSM-IV formulation was arbitrary and does not capture many patients who present in primary care settings [13,14]. Others argue that the distinction between subcategories of somatoform disorders and differences between somatoform disorders and functional syndromes is unclear and/or unjustified [14,26,30]. In addition, patients often judge the diagnosis of a somatoform disorder as being 'unacceptable’ (possibly due to expected stigmatisation) so physicians may prefer to diagnose a related functional disorder such as chronic fatigue [21]. Although functional diagnoses may be more immediately appealing, they may hinder the development of appropriate treatment strategies in some cases.

Another subtype of unmodifiable barriers to diagnosis may include other practical constraints such as a lack of consultation time in primary care settings. For example, previous research has suggested that physicians do not document as many symptoms as patients report in questionnaires [16,31] which may preclude follow-up investigations in future visits. With infinite time, physicians would be able to effectively gather an in-depth account of the patient’s symptomatic and psychosocial circumstances and rule out any organic cause of the unexplained symptoms. Physicians are aware of these constraints and it is highly likely that this affects their management of the patient [22]. Another role of the physician when managing patients is to legitimise work absenteeism or other social benefits [22,25,26]. Whether and how this role interferes with diagnosis, however, is unclear.

### Aims and objectives

The current review will systematically interrogate the published literature with the aim to determine the current factors that prevent the successful diagnosis of somatoform-type disorders. This will involve a both a description of the barriers and a documentation of their frequency reported in the literature. It is only when the barriers to successful diagnosis are known, that they can be addressed in training programmes, policy development and other healthcare improvement initiatives. By combining information from multiple sources which employ qualitative and quantitative techniques, we believe our review will provide great insight into the current situation.

In the process of our review, we will also be able to recognise the differences in methodological techniques, recommend potential avenues for future research and comment on the literature in this field as a whole. We will also have the opportunity to suggest new strategies to improve patient-centred care based on the synthesis of the results. Ultimately, we hope this information can be used to improve the diagnostic process and the treatment of such disorders.

## Methods/Design

The review will be conducted in one stage which synthesises the information in depth. The aim of this review is to gain insight into the diagnostic barriers in primary care in general; therefore, no subgroup analyses are planned. The method and reporting of the review will adhere to the guidelines outlined in the PRISMA statement, wherever possible [32,33]. However, it is unlikely that summary measures, any meta-analytical analyses and bias assessment of randomised control trials will be relevant to the current review, given the qualitative nature our questions. The review has been registered in an international registry of systematic reviews PROSPERO (CRD42013002540).

### Search strategy for the identification of relevant studies

The search strategy was developed to include four main keywords (and their synonyms). Specifically, each of the four keyword’s ((1) barrier, (2) primary care, (3) diagnosis and (4) somatisation) synonyms were combined using the logical operator OR. The four search strings were combined at the end using the logical operator AND. Wildcard operators were used to make the search more lexically flexible and to encompass differences between UK and US spelling. Please see Additional file 1 for full details.

The literature search will include records found from the following four databases: EMBASE (OVID interface); PsycINFO (OVID interface); MEDLINE (OVID interface); and the Cochrane Database of Systematic Reviews.

The search strategy will remain the same across the databases, but the list of to-be-searched fields and search formulation will differ slightly. The titles in the reference lists of included studies will also be reviewed so that additional relevant studies can be included. In order to concentrate on studies which reflect the current barriers in clinical practice, and limit the scope of the study for pragmatic reasons, we will only include studies from the past 10 years. The review will also only include studies which are published in English and German in peer-reviewed journals. Although these limits may potentially bias the results of the review, we argue that the most recent studies in English and German represent a large part of the forefront of this research field. Future research should specifically investigate cultural differences in the diagnosis and management of somatoform disorders and whether diagnostic behaviour has changed over time. No other functional or conceptual limits will be imposed [34].

### Criteria for inclusion and exclusion of studies in the review

Our initial eligibility criteria relating to the type of studies that can be included were two-fold: (1) studies that have undergone a typical peer-review process; and (2) articles which contained original data or were a systematic review which roughly corresponded to a level of evidence greater than five in the Centre for Evidence-Based Medicine’s schema [35]. Due to the qualitative nature of our question, we will not discriminate between quantitative or qualitative data. Although the synthesis of such heterogeneous research will be difficult, we believe this approach will be the best to comprehensively answer our question.

The included studies or systematic reviews must be based on patients with somatoform or functional symptoms in primary care, that is, before being referred to a specialist medical or psychological/psychiatric practitioner. We will not exclude patient populations based on age, gender, ethnicity or other such demographic variables. More specific inclusion and exclusion criteria may also be modified during the initial stages of design. A full list of the exclusion criteria is given in Additional file 2.

Two reviewers will independently review studies according to the attached inclusion–exclusion criteria during both the initial screening of studies (with abstracts and titles) and the investigation of full texts in the second round. Differences in opinion about the eligibility of the study will be discussed. If, after a discussion, no agreement as to the inclusion/exclusion of the study can be made, a third reviewer will be consulted. When both reviewers agree there is insufficient information to reject a paper in the initial round, it will be included in the second (full-text) round of eligibility assessment. Inter-rater reliability coefficients will be calculated to assess the agreement between the two independent reviewers when assessing the eligibility of full text studies. In addition, the number of publications (percentages) which needed the input of a third reviewer after discussion will be reported.

### Information management

Records identified through the literature searches will be managed using a custom-designed database in Microsoft Access. The initial list will be exported from the OVID search interface or the Cochrane Database of Systematic Reviews as a text file or Microsoft Excel sheet and then imported to Access and modified accordingly. A custom-made form will display the bibliographic information about each record (including the abstract) and will contain text boxes where the reviewers can make comments, select any exclusion criteria and code the relevant information for each part of the extraction protocol (please see Additional file 3). Both reviewers will have their own copy of the database. Additional records identified from reference lists of included papers or from contact with corresponding authors will be entered into the database manually.

When it appears that there are multiple publications resulting from the same dataset, the reviewers will only include the most recent version. If any study details are unclear, the authors will correspond with study investigators for clarification.

### Description of methods and outcomes of the component studies

There are many published reviews which outline some of the problems with diagnosing somatoform disorders in primary care, however, many of these are based on expert opinion and are unsystematic [13,14,25,26]. Although useful in some contexts, editorials, unsystematic reviews, recommendation articles, overviews, commentaries and perspectives may be biased and will not be included in the current review. Individual case studies will also not be included because they may not necessarily reflect the general barriers that physicians face during diagnosis of somatoform-type disorders. We expect that the relevant studies will be largely observational using case-control or cohort study designs. However, some secondary outcomes of other types of original studies may also be relevant for the review. For example, a trial evaluating the efficacy of a physician education program may also include measures of physician attitude and confidence in diagnosis. In cases where the investigation of barriers to diagnosis is not the primary focus of the study, it will be up to the independent reviewers to decide whether the results are relevant to the review.

### Details of study coding and extraction of data

Two independent reviewers will review the titles and abstracts of publications after the list of eligible publications is compiled and duplicates are removed. These reviewers will use standardised inclusion/exclusion criteria (see Additional file 2) to assess the initial eligibility of publications. Clear reasons for inclusion/exclusion will be recorded using a custom-made database form and coded. It is sufficient in this preliminary stage that both authors agree to the inclusion or exclusion of studies; the exact code does not have to be the same for the two reviewers.

Full manuscripts will be retrieved for the papers which passed the initial round or when there is insufficient information in the title and abstract to exclude the study. During the second round, two authors will independently extract the relevant data from the manuscripts using a piloted, standardised form [36]. Studies which were reviewed during the pilot stage will be re-reviewed to ensure consistency throughout the review process. Results will be compared and discussed and the combined data should be entered into a new version of the database by both reviewers together. In this second stage, the primary reason for exclusion must be discussed, coded and agreed upon. The decision from each independent reviewer whether to include or exclude a study will also be recorded.

Data that will be extracted from each study includes (a more detailed protocol is provided in Additional file 3):

(1) Study characteristics - design, details of the healthcare setting, and so on

(2) Patient and practitioner characteristics - demographic variables, diagnostic and relevant medical history

(3) Nature or reason for the consultation (symptoms presented by the patient)

(4) The diagnostic process (what diagnosis was given, description of the information exchange, what clinical and further examinations where made, the diagnostic framework used, whether patient and doctor discussed psychological distress)

(5) Any further potential problems (other relevant interpersonal or societal factors)

(6) Study information which is relevant to the assessment of bias, methodology or the quality of the study. This will also include an assessment of the level of evidence that can be obtained from the study [35]

### Procedures employed to evaluate, synthesise and classify information

The first step in the synthesis and evaluation of studies is to classify each study as quantitative or qualitative. The level of evidence gained from the study will also be assessed as per the schema from the Oxford Centre for Evidence-Based Medicine [35]. Details of the included studies will be presented in a table similar to Table 1. This will include the authors, type of study, the characteristics, diagnostic framework used and the quality assessment.

**Table 1:** Details of to-be-included studies with a fictional study example

**Study authors (year)**	**Type of study**	**Study characteristics**	**Diagnostic framework used**	**Quality assessment**
For example, Murray et al. (XXXX)	Qualitative follow-up of RCT study	15 GPs interviewed about difficulties in diagnosing somatoform patients	DSM-IV	Sound methodology: thematic analysis coded independently

For quantitative studies, two reviewers will independently assess the risk of bias by using elements from the Cochrane Collaboration’s tool [37,38]. Depending on the nature of the design, the two reviewers will assess the following possible sources of bias: selection bias, performance bias (of participants and/or study conductors), detection and measurement bias (of outcome assessors), attrition and exclusion bias, reporting bias and any other relevant source of bias. The risk of these biases will be assessed as either: 'High’ (meaning that there is a high risk of bias), 'Low’ (meaning that there is a low risk of bias), 'Unclear’ (it is unknown whether this bias is present or not) or 'N/A’ (meaning that this type of bias is not applicable to this type of study).

In contrast, there are different approaches to assess the validity and relevance of qualitative studies and the relative utility of each approach is debated [39-42]. Wherever possible, we will adhere to the recommendations of the Cochrane Collaboration, who argue it is important to evaluate the degree of researcher bias, quality of reporting, methodological rigour and the conceptual depth of the studies [43,44]. Specifically, this can be done by assessing the: (1) credibility (the extent to which the data fits the views of the participants); (2) transferability (the possibility that the results can be transferred to other settings); (3) dependability (the logic and clear documentation of the research); and (4) confirmability (the extent to which the analysis is directly based on the data and the reflexivity of the researchers), of the study [43]. Any studies that show a clear violation of any of these aspects of quality will be excluded. If needed, a well-established checklist will be used to evaluate the clinical effectiveness of each qualitative study once the final list of studies is available [45]. In addition, the reviewers may identify additional sources of bias which are commonly observed in the literature [46,47].

The heterogeneity of the possible studies to be included in the review means that the synthesis of information will be difficult. Given that we aim to be as comprehensive as possible, we will undertake a multilevel approach to the synthesis. In this way, the qualitative and quantitative information will be presented separately and then combined within the same review [44]. Based on the recommendations of a recent review by Barnett-Page and Thomas [48], we propose that a combination of a textual narrative and thematic analysis approaches to data synthesis would be the best for our study. This method has advantages when extracting and synthesising information of different types [48-50]. The results of such designs can have clear recommendations for future action, for example, to improve policy or training programs [48]. One of the challenges associated with this approach, however, is that there may be no overall theoretical structure in which we can place our results [50]. Our aim, however, is to aggregate the perceived barriers and not necessarily to test or develop a theoretical model. We have developed a preliminary conceptual framework within which we will organise the results (Table 2). The barriers are first separated according to whether they are modifiable or non-modifiable. The modifiable barriers are then split into patient-, doctor-related and demographic factors whereas the non-modifiable factors are divided into conceptual barriers, operational barriers and other practical constraints.

**Table 2:** The conceptual framework including various modifiable and non-modifiable barriers

		**Identified barrier**	**Studies from Table**1**which identified this barrier**
Modifiable barriers	Patient-related barriers	e.g. fear of stigmatisation	
	Doctor-related barriers	e.g. fear of missing a somatic disease	
	Demographic factors	e.g. gender or ethnicity stereotypes may hinder diagnosis	
Non-modifiable Barriers	Conceptual barriers	Definition of somatoform disorder is unclear	
	Operational barriers	Diagnostic criteria are too strict	
	Other practical constraints	Lack of time in consultations	

Given the likely high variability of the to-be-included data, we will most likely need a number of iterations in the extraction and grouping process. It may be advantageous to use a separate software package to assist in this process such as QARI or ATLAS.ti [50].

Once all the data regarding the barriers to diagnosis, recommendations for future action and study characteristics are extracted from the individual publications, a thematic analysis will be undertaken [49]. Extracted data from the combined database from both reviewers will be then clustered and coded according to themes by both reviewers using elements of previously established approaches [41,42,50]. Although this approach is labour-intensive, it is considered to be critical to the success of the review [41]. Reviewers will then independently group these themes into higher order topics and a discussion will result in the final higher-order themes [42]. Additional information about the study design or other methodological considerations will be noted. We argue this is important to minimise conclusions being based on studies which are individually unreliable. Similarly to a textual narrative synthesis, this will allow the reviewers to identify and comment on gaps or problems in the literature [49].

### Timeframe

**Figure 1:**
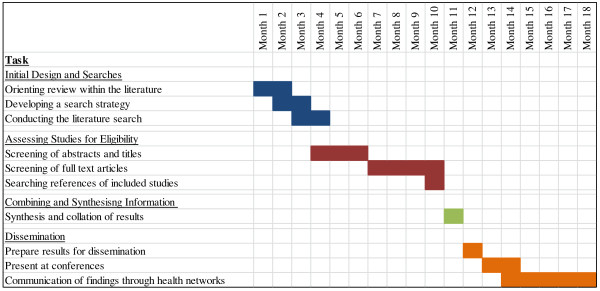
**Gantt chart depicting the expected time frame for the development of the review.** The major tasks (underlined) and subtasks within the review process are shown on the left-hand side. On the right-hand side, the expected duration of each stage is depicted by coloured boxes which correspond to the month within the whole project.

The proposed timeline for the conduction of the review can be seen in Figure 1. We anticipate that the review should take approximately 1 year from inception to completion. Once the results have been compiled, any clinical recommendations will be communicated to healthcare networks and scientific conferences during the following 6 months. In the greater Hamburg metropolitan region, there are already such networks established which should facilitate the application of any results from the review into clinical practice (for example, Sofu-net: Network for somatoform and functional disorders [51]).

## Discussion

Underdiagnosis and a lack of research into somatoform-type disorders is a problem that is widespread in primary care [12]. It is, therefore, important to identify barriers to successful diagnosis in order to design effective intervention programmes. Although the results may suggest some potential intervention targets, the specific types of interventions are beyond the scope of this review and should be directly investigated in future research.

Given the changes to the criteria of somatoform-type disorders in DSM-5 [17] it will be important to investigate whether changes in the diagnostic criteria alleviate some of the current problems associated with diagnosis. We intend, therefore, to update the review to reflect what changes, if any, the new criteria had on the diagnosis of somatoform disorders in primary care in the future and whether, care for patients with somatoform or functional symptoms is improved.

## Endnotes

^a^Differences in estimations depend on the sample and diagnostic criteria.

^b^Although a lot of research has predominantly focused on DSM criteria, many criticisms of the classification of these disorders are also relevant to the International Statistical Classification of Diseases and Related Health Problems (ICD).

## Competing interests

This work is being undertaken by the Department of Psychosomatic Medicine and Psychotherapy within the University Medical Center Hamburg-Eppendorf and is not externally funded by any third party bodies. The authors are not aware of any conflicts of interest which may affect the results of the review or protocol.

## Authors’ contributions

AMM, AA and BL formed the original research question and search strategy. AMM and AA conducted the initial screening of results. AMM and AT developed the approach to data extraction and synthesis. BL provided intellectual input at each stage of design and supervised the project. AMM drafted the manuscript and all authors provided feedback, read and approved the final manuscript.

## Authors’ information

AMM and AT are post-doctoral researchers working at the department of Psychosomatic Medicine and Psychotherapy, University Medical Center, Hamburg-Eppendorf and Schön Clinic Hamburg-Eilbek. AA is now working in the department of General Medicine, University Medical Center, Hamburg-Eppendorf. BL is the Director (W3 Professor) of the department of Psychosomatic Medicine and Psychotherapy, University Medical Center Hamburg-Eppendorf and the Head Physician of the University Hospital of Psychosomatic Medicine and Psychotherapy, Schön Clinic Hamburg-Eilbek.

## Acknowledgements

We kindly acknowledge the helpful suggestions of the somatoform disorders workgroups and Dr. Björn Riegel during the development process of the protocol.

## Supplementary Material

Additional file 1 **Search strategy.** Search strategy to search MEDLINE, PsycINFO, EMBASE and the Cochrane Database of Systematic Reviews. Click here for file

Additional file 2 **Protocol for the inclusion/exclusion of studies.** Additional file 2 is a document which specifies the inclusion/exclusion criteria and their codes. Click here for file

Additional file 3 **Protocol for the Extraction of Data.** Additional file 3 is a document which outlines the data which should be extracted from full-text manuscripts. Click here for file
