# Supplementary material for: Barriers to the diagnosis of somatoform disorders in primary care: protocol for a systematic review of the current status
Source: Syst Rev. 2013 Nov 8;2:99. doi: 10.1186/2046-4053-2-99 (PMC3830509; doi:10.1186/2046-4053-2-99)
Supplement: Additional file 1 — Search strategy. Search strategy to search MEDLINE, PsycINFO, EMBASE and the Cochrane Database of Systematic Reviews. [file 2046-4053-2-99-S1.docx]

**Additional file 1:**

Search strategy using the OVID interface to search MEDLINE, PsycINFO and EMBASE concurrently.

(1) (Barrier* or difficul* or hurdle* or hindrance or hinder* or imped* or trouble or complicat* or reluctan* or challeng* or problem* or prevent* or obstacle* or issue* or resist* or obstruct*).mp.

(2) (primary care or general care or family care or primary patient or general patient or general practic* or general practit* or family practic* or family practit* or primary practic* or primary practit* or GP or GPs or family doctor* or primary doctor* or general physician* or family physician* or primary physician* or family clinic* or general clinic* or primary clinic* or primary healthcare or primary health care or general health care or general healthcare or family health care or family healthcare or family medic* or general medic* or primary medic*).mp.

(3) (diagnos* or underdiagnos* or misdiagnos* or recogni* or asses* or identif* or acknowledg* or detec* or evaluat*).mp.

(4) (somati#ation or somatoform or psychosomatic dis* or medically unexplained or functional somatic symptom* or functional symptom* or functional syndrome* or functional somatic syndrome* or Conversion disorder* or Hypochondri* or Body dysmorphic disorder* or Pain disorder* or multiple unexplained symptoms or MUS).mp

(5) (1) AND (2) AND (3) AND (4)

The abbreviation .mp relates to a “multipurpose” search using the Ovid interface which searches a variety of fields for the given search terms such as title, abstract and keywords. The exact fields, however, differ slightly according to the specific database.

The search strategy will be slightly modified when searching the Cochrane Database of Systematic Reviews.
