# Supplementary material for: Barriers to the diagnosis of somatoform disorders in primary care: protocol for a systematic review of the current status
Source: Syst Rev. 2013 Nov 8;2:99. doi: 10.1186/2046-4053-2-99 (PMC3830509; doi:10.1186/2046-4053-2-99)
Supplement: Additional file 2 — Protocol for the inclusion/exclusion of studies. Additional file 2 is a document which specifies the inclusion/exclusion criteria and their codes. [file 2046-4053-2-99-S2.docx]

**Additional file 2:**

Protocol for the inclusion/exclusion of studies:

**Inclusion Criteria:**

Studies were included in the review if:

1. The study focused on the diagnostic process or barriers to the diagnosis of somatoform disorders in primary care settings.

**Exclusion Criteria:**

Studies were excluded from the review if they were judged to belong to one of the following categories:

1. Somatization or somatization scores were considered only as a risk factor for another specific phenomenon or condition or the focus is to determine the predictors of somatization e.g. sexual abuse.
2. The focus of the research is to discuss psychological or psychiatric treatment, management or intervention approaches (of either somatoform disorders or other conditions).
3. The aim of the research is to validate current diagnostic instruments in different languages or samples.
4. The focus of the research is to investigate multi-morbidity or prevalence patterns in primary care settings or particular population groups.
5. The focus of the research is to improve or discuss the diagnosis of non-somatoform disorders or psychological problems generally or other phenomena e.g. depression and anxiety disorders or suicide.
6. The focus of the research is to examine the use of medical services or to reduce the use of medical resources/costs or participation restriction (e.g. work absenteeism) or to discuss the structure of health care systems.
7. Other (specify). For example the focus of the article is: Profile of somatoform patients (describing the characteristics of the typical patient), cultural differences in somatoform disorders generally, factors which affect disclosure and referral behaviour and when it is clear that the diagnosis does not take place in primary care settings.

98. The abstract (study) does not relate to a peer-reviewed journal article or review in a peer-reviewed journal.

99. The language of the article was not English or German.
