# Supplementary material for: Barriers to the diagnosis of somatoform disorders in primary care: protocol for a systematic review of the current status
Source: Syst Rev. 2013 Nov 8;2:99. doi: 10.1186/2046-4053-2-99 (PMC3830509; doi:10.1186/2046-4053-2-99)
Supplement: Additional file 3 — Protocol for the Extraction of Data. Additional file 3 is a document which outlines the data which should be extracted from full-text manuscripts. [file 2046-4053-2-99-S3.docx]

**Additional file 3:**

Protocol for the Extraction of Data

**Study Details:**

Please check that the study details (authors, title and first line of the abstract) correspond with the pdf documents.

***Original Data and Systematic Review Radio Buttons:*** 🔾 Please select the appropriate option if the study contains original data (or not) and if is a systematic review (or not).

***Type of Study Comment Box:*** In the comment box, please give a comment as to the nature of the study by adding a comment.

**Extraction of Study Information:**

(1) *Study Characteristics* (design, details of the health care setting etc.), e.g. 49 Primary Care Settings in Rural Australia.

(2) *Patient and General Practitioner Characteristics* (demographic variables, diagnostic and relevant medical history)

(3) *Nature or reason for the Consultation* (e.g. symptoms presented by the patient)

(4) *The Diagnostic process* (what diagnosis was given, description of the information exchange, **whether patient and doctor discussed psychological distress**)

(5) *Barriers to the diagnosis* 1-4 (what problems or hindrances were identified in the process of diagnosis e.g. negative GP attitude towards somatoform disorders, patients want to prioritise attention towards physical symptoms). Please include only one barrier per field and any remaining barriers can be entered into the “*Other barriers*” box.

(6) *Any further Potential Problems* (any other reasons (interpersonal or societal) or contributing factors which may adversely affect the diagnosis of somatoform-type disorders in primary care). E.g. it may not be considered masculine for men in rural Australia to discuss psychological distress.

***Exclude Radio Buttons:*** 🔾 Please select the appropriate radio button of either “include” or “exclude” to determine whether the paper should be included in the review. At this stage we must agree on the reason to exclude any papers and if an agreement cannot be reached a third reviewer may be required. Please note the reason for exclusion in the corresponding box.

**BIAS:**

Please rate whether there is HIGH, LOW or an UNCLEAR risk of bias for each subsection. Then, when relevant, please extract the relevant sentence from the article as a record of the appropriate part of the article corresponds to that type of bias. Any potential additional biases should be added into the box “Other sources of bias”. Please record any methodological issues of the study here.

**Level of Evidence:**

Drop-down menu: Please use the drop-down menu to select the appropriate Level of Evidence According to the Levels of Evidence guide from the Centre for Evidence Based Medicine (CEBM) (www.cebm.net). Please note the justification for this in corresponding box.

**Comments Round 2:** Please use this space to note any other relevant information about the article.
